# Overwintering honeybees maintained dynamic and stable intestinal bacteria

**DOI:** 10.1038/s41598-021-01204-7

**Published:** 2021-11-15

**Authors:** Peng Liu, Yujie Zhu, Liang Ye, Tengfei Shi, Lai Li, Haiqun Cao, Linsheng Yu

**Affiliations:** 1 College of Plant Protection, Anhui Agricultural University, Hefei, Anhui Province China; 2 College of Animal Science and Technology, Anhui Agricultural University, Hefei, Anhui Province China

**Keywords:** Bacteria, Microbial communities

## Abstract

Honeybee is an important pollinator for maintaining ecological balance. However, scientist found the bizarre mass death of bees in winter. Meanwhile, some reported that the differences composed of intestinal bacteria between healthy honeybees and CCD honeybees. It is essential that explored dynamic changes to the intestinal bacteria in overwintering honeybees. We collected bee samples before overwintering, during prophase of overwintering, metaphase of overwintering, anaphase of overwintering, telophase of overwintering, and after overwintering. By using high-throughput sequencing targeting the V3−V4 regions of the 16S rDNA, the abundance of the intestinal bacteria were analyzed in overwintering honeybees. A total of 1,373,886 high-quality sequences were acquired and Proteobacteria (85.69%), Firmicutes (10.40%), Actinobacteria (3.66%), and Cyanobacteria (1.87%) were identified as major components of the intestinal bacteria. All core honeybee intestinal bacteria genera, such as *Gilliamella*, *Bartonella*, *Snodgrassella*, *Lactobacillus*, *Frischella*, *Commensalibacter*, and *Bifidobacterium* were detected. The abundance of Actinobacteria, *Bartonella*, and *Bifidobacterium* increased initially and then decreased in winter honeybees. There were no significant differences in the richness and evenness of the microbiota in overwintering honeybees; however, there was a statistically significant difference in the beta diversity of the intestinal bacteria after overwintering compared with that in other groups. Our results suggested that honeybees maintained their intestinal ecosystem balance, and increased the abundance of gut probiotics in response to environmental and nutrition pressures in winter.

## Introduction

Honeybee (*Apis mellifera*) belongs to the Apidae family and the *Apis* genus. It is widely distributed all over the world. As a pollinator, honeybees are important insects. They play a crucial role in maintaining the ecological balance worldwide via pollination and are valuable economic resources for crop and fruit tree pollination, hive production, and apitherapy^1–4^. Honeybees collected pollen, water, nectar and gum and monitoring contributes to the ecological impact statement for heavy metals, fungicides and herbicides, and help to chart environmental health maps^5,6^. In the early 21th century, scientist found many hives contain a complete absence of adult bees, including a large number of dead adult bees, and the adult bees vanish during winter^7^. The phenomenon is termed colony collapse disorder (CCD) and mainly happens in winter^8^. As the number of honeybees continues to decline, many reports suggested that CCD was multifactorial, being affected by chemical agents, pathogenic organism, innutrition, and habitat loss^9–12^. Cox-Foster et al. found there was a significant difference in the abundance of members of the intestinal bacteria between a healthy hive and CCD hive. It suggested that the intestinal bacteria might play an important role in honeybees in CCD hives^13^.

Microorganisms and hosts are mutually selective and show co-evolution based on symbiotic principles. The intestinal bacteria are established from ‘neutral’ to beneficial to essential in hosts^14^. Intestinal microbial community of bees has formed a close relationship with their host though a long evolutionary process. Honeybees harbor a relatively simple but remarkably specialized and consistent intestinal microbial community. The intestinal bacteria of western honeybees (*Apis mellifera*) mainly comprises nine species, including gram-negative bacteria (*Gilliamella apicola* and *Frischella perrara*), Betaproteobacterium (*Snodgrassella alvi*), *Lactobacillus* (Firm-4 and Firm-5), Bifidobacterium (*Bifidobacterium asteroides* and *Bifidobacterium coryneforme*), Alphaproteobacteria (Alpha-1 and Alpha-2)^15–17^. A healthy and stable intestinal bacteria are vital for the beneficial functions of honeybees. The intestinal bacteria help the host to digest food and provides essential nutrients, include vitamins and carbohydrates^18^. Some gut bacteria can also help hosts to detoxify harmful molecules and protect against pathogens and parasites^19,20^. The beneficial bacteria resist colonization and development of pathogens and parasites by producing antimicrobial compounds and synthesizing key components of the locust cohesion pheromone^16^. During the growth stage of honeybees, the intestinal bacteria are also involved in growth improvement and immunomodulation of its hosts^15,19^. The intestinal bacteria of a healthy honeybees are characterized by its dynamic stability^21^. However, it can show dysbiosis or disequilibrium when challenged by many factors alone or combination, such as a different diet^22^, pesticide exposure^23^, parasite and pathogen infection^24^, and behavioral tasks^25^.

In winter, honeybees should undergo a special reaction to deadly cold. While resources are limited and flying constrained, the numbers of healthy overwintering honeybees are critical for colony health and survival. Honeybees are stressed by nutritional deficiency and the poor environment in winter. Honeybees feed strictly on food stores (pollen, beebread, and honey) and form a tight cluster for thermoregulation by consuming muscle energy inside the hive^26^. Although honeybees stay in the hive all winter, the hive also loses many individuals. Meanwhile, honeybees are fragile in winter and are easily infected by parasites and pathogens^27^. It is a huge challenge for the hive to maintain enough honeybees. Surprisingly, honeybees had lower immunity and lower nutrition in winter compared with those in summer, but winter honeybees had the longest length of life, living for 150–304 days in winter^28–32^. This far exceeded the length of life in spring, summer, and autumn^28,29,33^. The function and role of intestinal bacteria in winter honeybees is unknown and the intestinal bacteria may play a crucial role in winter honeybees. Previous studies have shown that there is an important interconnection between insulin/insulin-like growth factor signaling pathway, lifespan, and the intestinal bacteria^19,34–36^. Compared with that during summer, the composition and structure of the intestinal bacteria were different and the overall bacterial load was about 10 × larger in winter honeybees^37^. However, in the long winter, it is unknown how the intestinal bacteria of honeybees changes. In the present study, we investigated the composition and structure of the intestinal bacteria at different time periods and explored the dynamic changes of the intestinal bacteria in overwintering honeybees.

## Results

**Figure 1:**
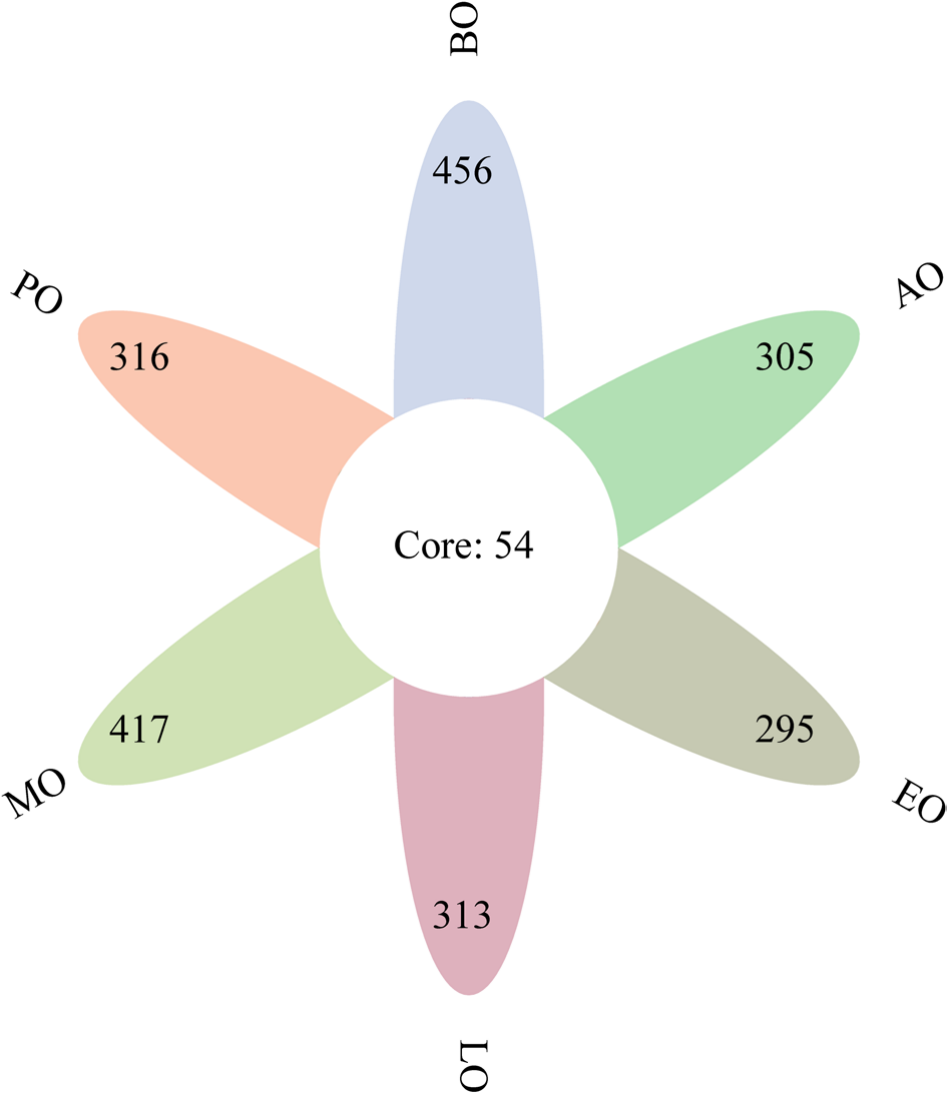
The features composition of the intestinal bacteria of honeybees in winter. The number in the figure is the number of features and different colors represent different groups.

To investigate the dynamic change in the gut microorganisms of honeybees in winter, 18 samples from a total of 270 honeybees were collected during two months in winter. Using 16S rRNA pyrosequencing based on the V3–V4 region (see Supplementary Table 1), a total of 1,374,167 high-quality sequences were acquired and the lengths of 99.98% of the effective sequences reads were between 400–500 bp. Most of the sequences were valid and only 0.141% had non-zero values. The sum of features number was 3014 and the average was 167 for the 18 samples. As shown in Fig. 1, the most features were found in the before overwintering sample (510 features), while the fewest features were found in the telophase of overwintering sample (349). Fifty features were common to all groups.

**Figure 2:**
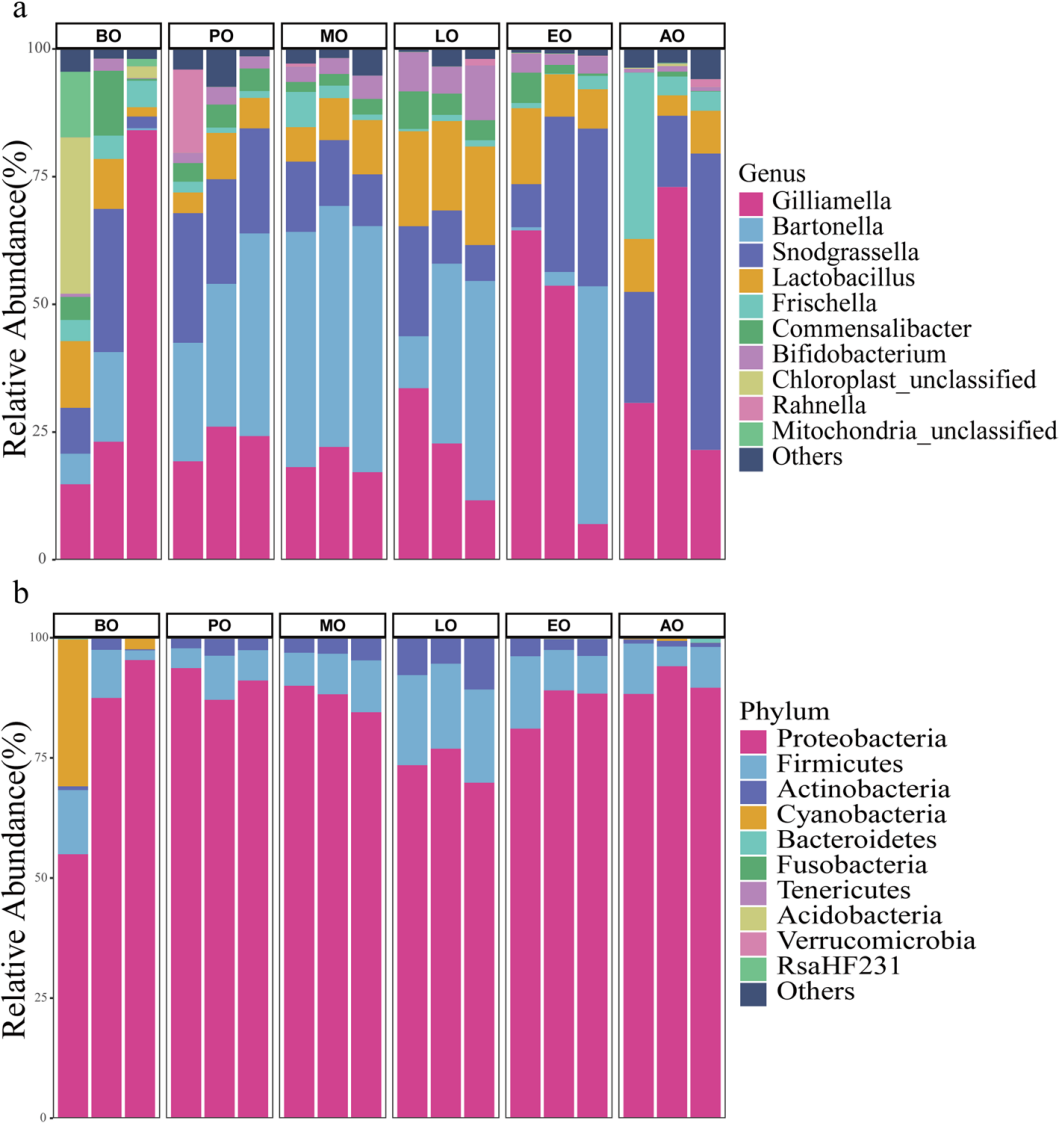
Relative abundance of the dominant gut bacterial communities. (**a**) Relative abundance of the dominant gut bacterial communities in honeybees at the phylum level. (**b**) Relative abundance of the dominant gut bacterial communities in honeybees at the genus level. Each bar represents the average relative abundance of each bacterial taxon within a group. The lowest color block of the column represents the highest abundance (biological repeat mean) of microorganisms in all samples.

**Figure 3:**
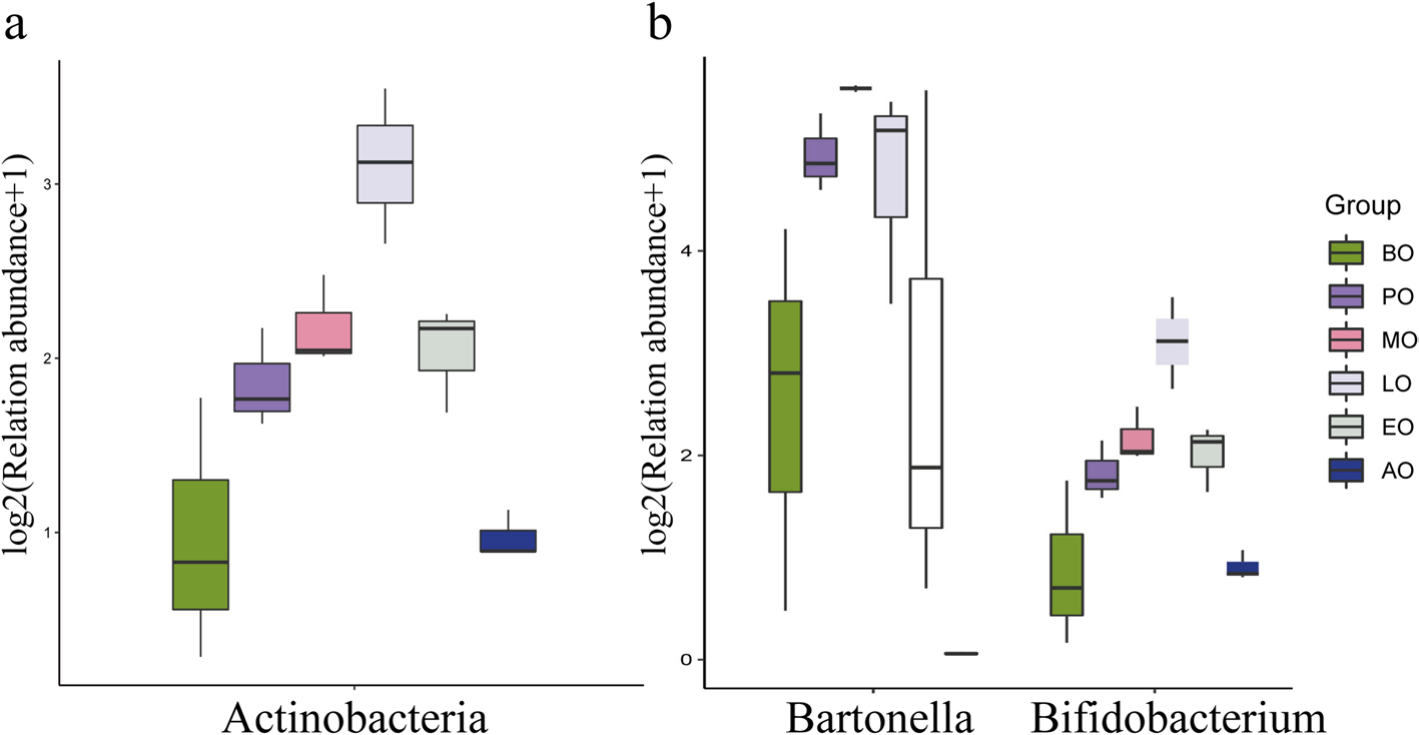
The significant differences in the bacteria in honeybees at the phylum and genus levels. (**a**) Box plot of significant differences in bacteria (Actinobacteria) at the phylum level. (**b**) Box plot of significant differences in bacteria (Bartonella and Bifidobacterium) at the genus levels.

Using QIIME2 default settings, the taxonomic distributions at the phylum and genus levels were summarized in Fig. 2. According to their average relative abundance in all groups, Proteobacteria (85.69%), Firmicutes (10.40%), Actinobacteria (3.66%), and Cyanobacteria (1.87%) were major components of the gut bacteria. The abundance of these core phylum was in excess of 97.9% in the honeybee gut. At the genus level, the abundance of the core bacteria was in excess of 93.3% in the honeybee gut, and *Gilliamella* (31.52%), *Bartonella* (21.92%), *Snodgrassella* (19.15%), and *Lactobacillus* (9.91%) had high abundance (> 5%). Other core gut bacteria were detected, including *Frischella* (4.20%), *Commensalibacter* (3.45%), and *Bifidobacterium* (3.17%). the abundances of *Bartonella* (Kruskal test, p = 0.02) and *Bifidobacterium* (Kruskal test, p = 0.02) were significantly different in winter honeybees. By analyzing the significant difference abundance of bacteria in genus and phylum level (Fig. 3), we found that the abundance of *Bartonella* increased from 7.97% in the before overwintering sample to 47.14% in the metaphase of overwintering sample, and then decrease to 0.04% in the after overwintering sample. The abundance of *Bifidobacterium* increased from 1.04% in the before overwintering sample to 7.88% in the anaphase of overwintering sample, and then decreased to 0.88% in the after overwintering sample. At the phylum level, the abundance of Actinobacteria was significantly different in winter (Kruskal test, *p* = 0.02). It increased initially from 1.14% in the before overwintering sample to 7.91% in the anaphase of overwintering sample, and then decreased to 0.05% in the after overwintering sample.

**Figure 4:**
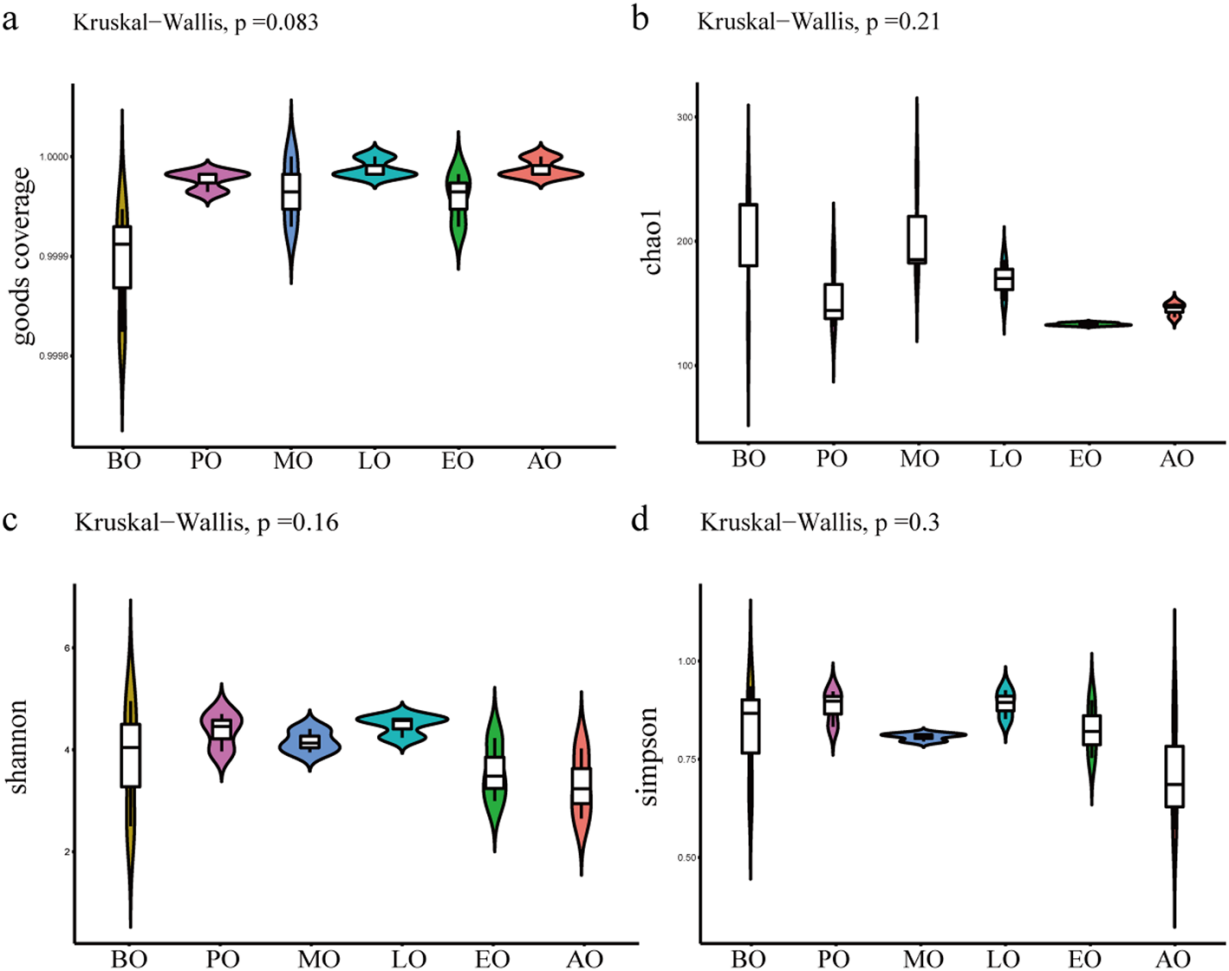
Alpha diversity of gut bacteria in winter honeybees. The amount of bacterial diversity was determined by comparing Goods coverage (**a**), the Chao 1 index (**b**), the Shannon index (**c**), and the Simpson index (**d**).

To assess the difference in gut microflora richness and evenness, four alpha diversity parameters, including the Chao 1 index, the Goods coverage index, the Shannon index, and the Simpson index were applied (Fig. 4). The Goods coverage index showed that the results of all groups represented the real situation of the samples. Analysis using the other three alpha diversity indices showed that there were no significant differences in richness and evenness among the six groups (*p* > 0.05).

**Figure 5:**
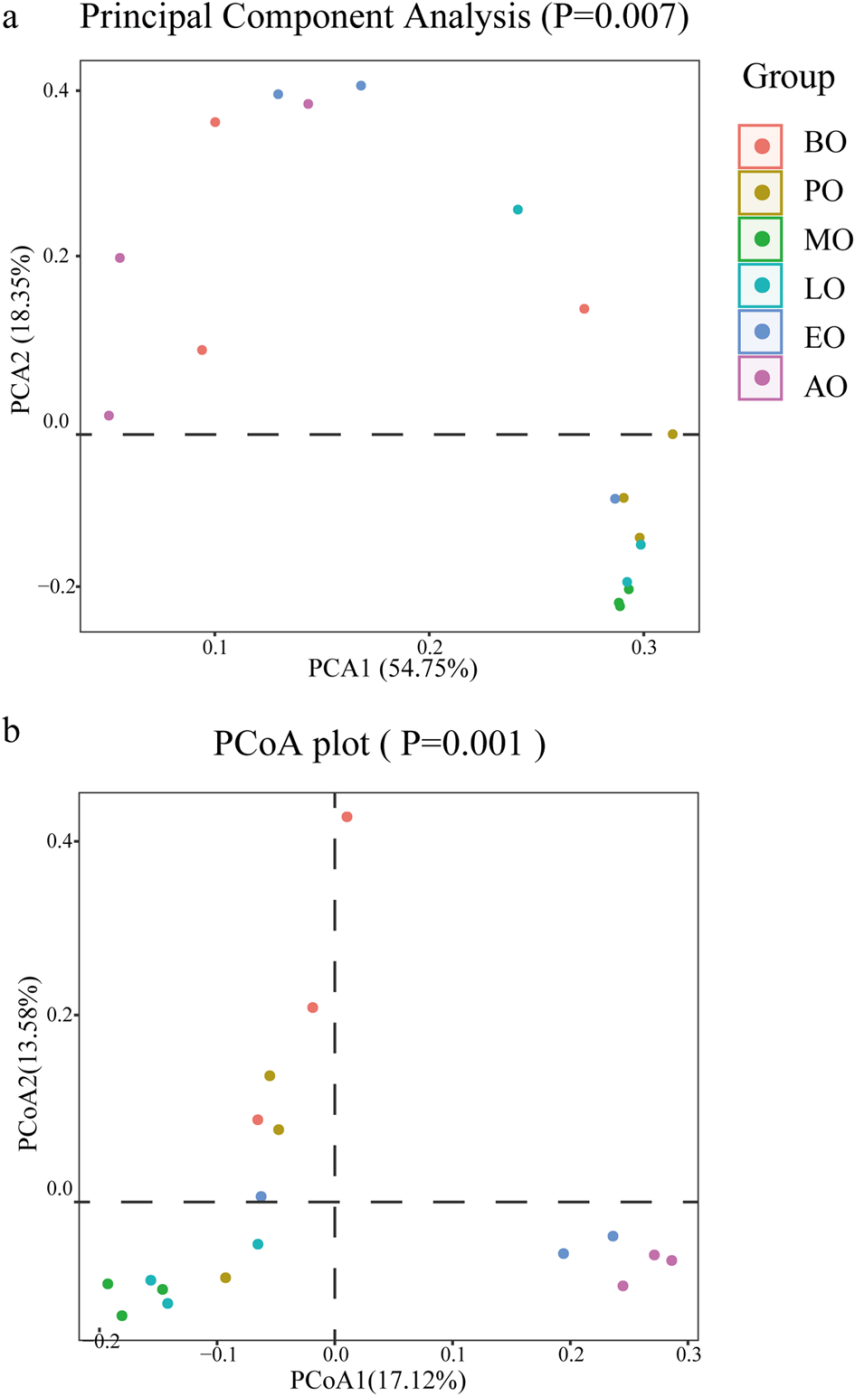
The PCA (**a**) and PCoA (**b**) plots of jackknifed unweighted UniFrac distances. Different colors represent different groups. Each axis explains the percentage of variation.

**Figure 6:**
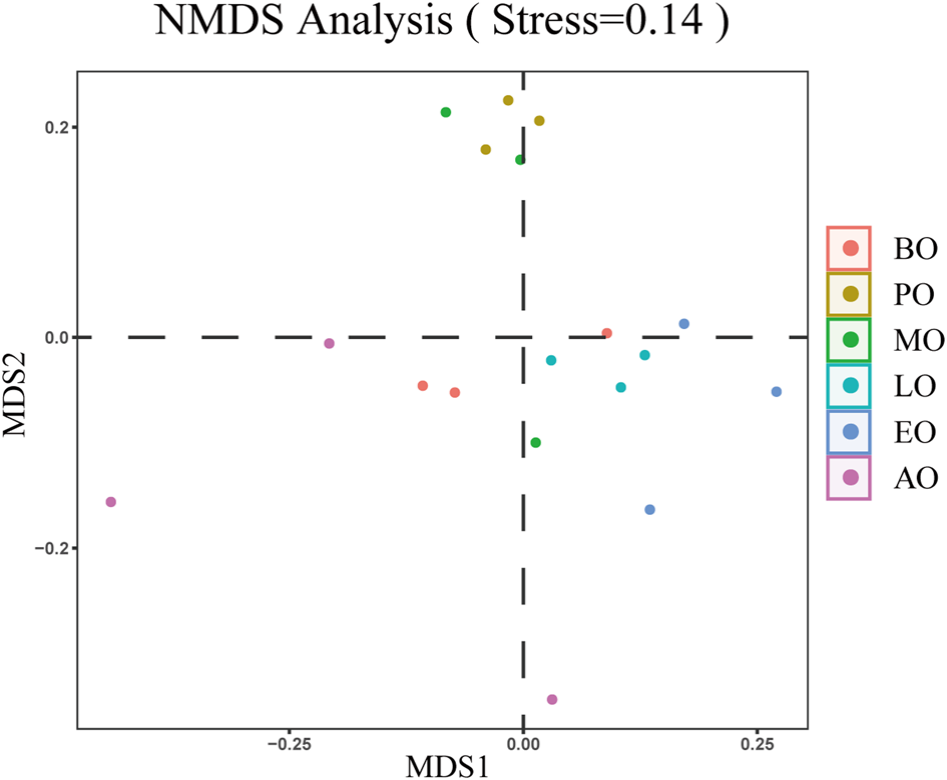
The result of Nonmetric multidimensional scaling analysis. The points in the graph represent the samples, the samples with different colors belong to different groups, and the distance between the points indicates the degree of difference between the samples.

For a more accurate estimate of the overall diversity or biological heterogeneity of the intestinal bacteria in winter, the beta diversity was assessed using principal component analysis (PCA) and principal coordinate analysis (PCoA) of unweighted unifrac distances (Fig. 5). The after overwintering honeybee samples were distinguishable from other samples. Consistently, ADONIS (an analysis of variance using distance matrices) on unweighted unifrac distances dissimilarities showed a statistically significant difference according to the groups (R^2^ = 0.42, *p* = 0.001). By permutational multivariate analysis of variance, analysis of similarities (Anosim), there was a significant distance between all samples (R = 0.47, p = 0.001). Moreover, nonmetric multidimensional scaling (NMDS) (Fig. 6) analysis of dissimilarities revealed a significant separation of samples, indicating that the communities of the after overwintering honeybees were different from those of the other samples. Integrating multiple methods, there was a difference in the beta diversity of the intestinal bacteria in the after overwintering sample compared with that of the other samples.

**Figure 7:**
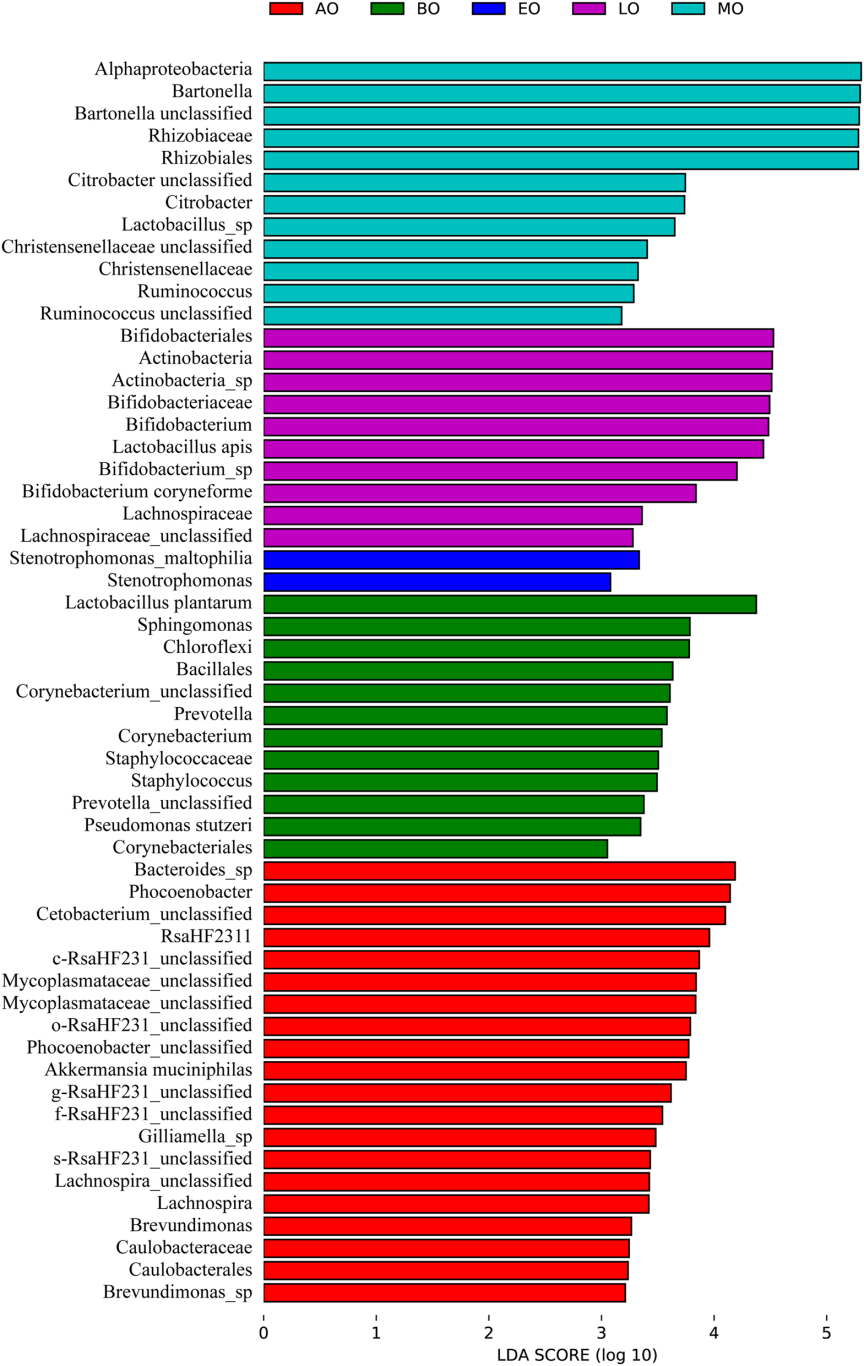
LEFSe analysis illustrating differentially abundant bacteria among samples with different haze levels.

To observe differences in the identified specific taxa in the intestinal bacteria, linear discriminant analysis (LDA) effect size (LEFSe) analysis was performed (Fig. 7). There was significant difference because the value of the logarithmic LDA score was bigger than 2. LEFSe analysis identified that Chloroflexi (class), Bacillales (order), Corynebacteriales (order), Staphylococcaceae (family), *Sphingomonas* (genus), *Staphylococcus* (genus), *Lactobacillus plantarum* (species), and *Pseudomonas stutzeri* (species) were rich in the before overwintering sample. Alphaproteobacteria (class), Rhizobiales (order), Rhizobiaceae (family), *Bartonella* (genus), and *Citrobacter* (genus) were rich in the metaphase of overwintering sample. Bifidobacteriales (order), Actinobacteria (phylum), Actinobacteria (class), Bifidobacteriaceae (family), *Bifidobacterium* (genus), and *Lactobacillus apis* (species) were rich in the anaphase of overwintering sample. *Stenotrophomonas* (genus) and *Stenotrophomonas maltophilia* (species) were rich in the telophase of overwintering sample. Caulobacterales (order), Caulobacteraceae (family), *Phocoenobacter* (genus), *Lachnospira* (genus), and *Brevundimonas* (genus) were rich in the after overwintering sample. There was no significant difference identified specific taxa in the prophase of overwintering sample.

## Discussion

The occurrence of CCD has made people aware of the importance of honeybees to the ecosystem. The decrease in the population of honeybees may cause ecological imbalance. The intestinal bacteria might play an important role in honeybees in CCD hives. Therefore, many studies have concentrated on the negative effects of the intestinal bacteria of honeybees, including the effects of abiotic and biotic factors^13,23,24^. The stability of the intestinal bacteria are important for the health of honeybees. The specialism of the intestinal bacteria and the social behavior of honeybees mark them as a simple model system for mechanistic studies on microbiota and bacteria-host interactions. In the evolutionary process, honeybees have formed a close relationship with intestinal bacteria. Many animals adapt cold environmental in winter by hibernation. Colony retains enough work bee and a queen in winter, and honeybees form a tight cluster inside the hive for adapt cold environmental^26^. Newly emerged worker bees are usually deprived of internal microbes and have few or no gut bacteria^15,38^. It's not conducive for honeybees to get through the bad environment and may be one of the reasons why queens do not lay eggs in winter. Previous studies have shown that there were significant differences in the intestinal bacteria of bees in winter compared with other season^37,39^. Meanwhile, honeybees may maintain the different ecological structure of intestinal bacteria in winter for overwintering. Winter represents a severe challenge to honeybees and hives. Therefore, in the present study we investigated the changes in the intestinal bacteria in winter honeybees based on 16S rRNA sequencing.

Our results showed that the nine core bacterial that normally comprise the honeybee gut bacterial community were present to differ degrees in overwintering honeybees. There was no significant difference in the diversity and microbiota community structures in overwintering honeybees. In addition, we did not find any pathogenic bacteria and opportunistic pathogens, such as, *Melissococcus plutonius*^40^, *Serratia marcescens*^41^, or *Hafnia alvei*^42^. These findings suggested that the gut bacteria of honeybees are stable and healthy during winter. Alteration of gut microbiota influences host metabolism and may affect the storage of energy in honeybees. Some studies had suggested that changes in diversity of gut microbiota are associated with some diseases and opportunistic bacteria^43^, included fungal pathogens and neogregarines^21^. This would benefit honeybees to reduce parasite and pathogenic infection for successful overwintering^24^. Although the overall diversity of the gut bacteria was stable, the distributions of some species changed during overwintering.

In the intestinal bacteria of honeybees, there are few bacteria in the midgut; however, there is a large bacterial community, comprising > 99% of the identified bacteria, in the hindgut^15^. The hindgut is divided into two discrete regions, the ileum and the rectum, which have distinct community compositions. The ileum is dominated by *Snodgrassella* and *Gilliamella* and has special structure peritrophic membrane^44,45^. Peritrophic membrane has very minute pores and anoxic environment that make a difficult entry passage for most the pathogenic, such as *Paenibacillus larvae*, it cannot penetrate through the peritrophic membrane^15,38^. In the ileum in winter honeybees, there was no significant difference in abundance of *Snodgrassella* and *Gilliamella*. This was not affect the function of peritrophic membrane and protected honeybees against pathogenic. Fecal waste is stored in the rectum, which might also function in the reabsorption of water and salts. The community in the rectum is dominated by the fermentative bacteria, *Bifidobacterium* and *Lactobacillus*^15,38^. As the most important genera of probiotics in the intestinal tract of honeybees^46^, *Lactobacillus* and *Bifidobacterium* play an important role in healthy of bees. It can inhibit other microorganisms on culture plates^47–51^, digest flavonoids and other compounds in the outer pollen wall and coat^52^, stimulates the production of host-derived prostaglandins and juvenile hormone derivatives^52^, release vitamins and short-chain fatty acids^53^. Short-chain fatty acids can also be absorbed by midgut cells as energy sources^53^. It can provide sufficient energy for bees to support individual heat release and colony temperature stability. This may be the reason for the abundance of some *Lactobacillus* species and *Bifidobacterium* significance higher during overwintering phase than before and after overwintering. Actinomycetes is an important microbial group in intestinal microflora that products bioactive compounds with antibiotic properties. Meanwhile, this group of bacteria was separated from brood cells, bees, and hive materials as well as natural sources in hives^54^. Actinomycetes may beneficial for maintain balance of intestinal and hives microflora by antibiotic compounds.

In this study, we observed that the rectum of the winter honeybees was expanded after the initiation of overwintering. This may provide an environment for increased bacterial attachment and growth. The rectum of winter bees contained more anaerobic microorganisms than the midgut^55^. Our results showed the anaerobic bacteria in overwintering honeybees was significantly different before and after overwintering. In the honeybee gut, anoxia is maintained by *S. alvi*, which forms a layer attached to the lleum wall, maintaining an anoxic gut environment^19^. The anaerobic bacteria were the main components in the honeybee rectum, and abundance of aerobic microorganisms were found lower than that of anaerobes, which was consistent with the results of a previous study^56^.

Before overwintering, foragers need to collect pollen, water, nectar, and gum from outside of the hive. This process allows honeybees to contact more bacteria, including pathogenic and opportunist bacteria. For example, the Phylum Cyanobacteria, which exists widely in water in the natural environment, was almost only found in the before overwintering sample^57^. Notably, after overwintering, the dominant species composition of the samples in winter honeybees was the same, and there was no significant difference in their abundance; however, there was a large difference in beta diversity, which was consistent with the results of a previous study^58^. Compared with other winter honeybees, honeybees defecated their waste and thus have a new intestinal environment after overwintering. Many bacteria that adhere to the gut wall and waste would be excreted into the natural environment. This behavior would cause the difference in beta diversity and intestinal structure.

## Material and methods

### Sampling of honeybees

To identify changes in the intestinal bacteria composition and abundance of honeybees (*Apis mellifera*) in winter (i.e., winter bees) across colonies, we selected a colony (Anhui Agricultural University, Hefei, Anhui province) that has successfully overwintered to spring reproduction for more than 3 years in normal management mode. According to the experience of colony management, honeybees begin to overwinter and stay in hive for about two months in Hefei, Anhui province, China. Taking this into account, we collected samples on the 10th of December, 2019 (before overwintering, BO), the 25th of December, 2019 (prophase of overwintering, PO), the 10th of January, 2020 (metaphase of overwintering, MO), the 25th of January, 2020 (anaphase of overwintering, LO), 10 February, 2020 (telophase of overwintering, EO), and the 25th of February, 2020 (after overwintering, AO). We sampled overwintering bees (PO, MO, LO and EO) on top of the frames inside the hive and before or after overwintering bees (include BO and AO) from the outside of the hive. A total of 18 samples containing 270 honeybees were collected. Each biological sample comprised 15 honeybees to decrease the effect of the differences between individual honeybees and three biological replicates were analyzed to decrease the experimental error.

Bees were anesthetized at − 20 °C for 5 min. Then, the surface bacteria of honeybees were cleaned off using 70% absolute alcohol, 80% absolute alcohol, 90% absolute alcohol, 0.1 M PBS (Phosphate-buffered Saline, purchased from Sangon Biotech, Shanghai, China), and sterile water^59^. After cleaning, the whole gut of the bees were carefully removed into 1.5 ml sterile centrifuge tubes using sterile forceps, immediately frozen using liquid nitrogen, and stored at − 80 °C until further analysis.

### DNA extraction

From each biological sample, 15 guts were pooled together into 1.5 ml sterile centrifuge tube. Total genomic DNA of intestinal bacteria was collected from guts sample using an E.Z.N.A. Stool DNA Kit (Omega Biotek, USA). Next, the intestinal bacteria 16S rRNA gene was amplified using PCR based on total genomic DNA and specific primers (Forward primer: 341F (5'-CCTACGGGNGGCWGCAG-3'; Reverse primer: 805R (5'-GACTACHVGGGTATCTAATCC-3'). The target V3 and V4 regions of the bacterial 16S rRNA gene were acquired^60^. The 25 μL PCR reaction mixtures containing 25 ng of total DNA, 12.5 μL of PCR Premix, 5 μL of primer, and PCR-grade water. The PCR conditions consisted of an initial denaturation at 98 °C for 30 s; 32 cycles of denaturation at 98 °C for 10 s, annealing at 54 °C for 30 s, and extension at 72 °C for 45 s; and then a final extension at 72 °C for 10 min. The purified and quantified PCR products was assessed on an Agilent 2100 Bioanalyzer (Agilent, USA) and with a Library Quantification Kit for Illumina (Kapa Biosciences, USA). The sequences of libraries were acquired by NovaSeq PE250 platform (Illumina, USA).

### Diversity analysis and statistical analysis

Based on the sequences of libraries, we obtained feature tables and feature sequences according to the fqtrim software (v0.94), Vsearch software (v2.3.4) and DADA2. Alpha diversity was applied to analyze the complexity of species diversity for a sample using five indices, including Chao1, Observed species, Goods coverage, the index Shannon, and the Simpson index. All these indices were calculated using QIIME2^61^. Beta diversity was also calculated using QIIME2, and the graphs of the analysis results by principal component analysis, principal coordinate analysis, Anosim, nonmetric multidimensional scaling and linear discriminant analysis effect size were drawn using the R package. BLAST was used for sequence alignment, and the feature sequences were annotated using the SILVA database for each representative sequence. Other diagrams were implemented using the R package (v3.5.2). In addition, we analyzed some data using charts created by by Microsoft Excel 2016 and using IBM SPSS Statistics v24 (IBM Corp., Armonk NY, USA). Certain images were constructed using GraphPad Prism 5 (GraphPad Inc., La Jolla, CA, USA) and edited using Adobe Illustrator CS6.

## Supplementary Information

Supplementary Information.

## Data Availability

All data generated or analysed during this study are included in this published article. All data that support the findings of this study have been deposited in National Center for Biotechnology Information (NCBI) Sequence Reads Archive (SRA) under Bio Project ID: PRJNA714595.
