# Supplementary material for: Overwintering honeybees maintained dynamic and stable intestinal bacteria
Source: Sci Rep. 2021 Nov 15;11:22233. doi: 10.1038/s41598-021-01204-7 (PMC8593070; doi:10.1038/s41598-021-01204-7)
Supplement: Supplementary file 1 — Supplementary Information. [file 41598_2021_1204_MOESM1_ESM.pdf]

Overwintering honeybees maintained dynamic and stable intestinal bacteria

Peng Liu[#], Yujie Zhu[#], Liang Ye[#], Tengfei Shi, Lai Li, Haiqun Cao, Linsheng Yu*

[#]These authors contributed equally to this work.

Author information

Affiliations

College of Plant Protection, Anhui Agricultural University, Hefei, Anhui Province, China

Peng Liu, Liang Ye, Tengfei Shi, Haiqun Cao & Linsheng Yu

College of Animal Science and Technology, Anhui Agricultural University, Hefei, Anhui Province, China

Yujie Zhu & Lai Li

contact information

Peng Liu, e-mail: 1042329682@qq.com

Yujie Zhu, e-mail: 343020289@qq.com

Liang Ye, e-mail: 623545372@qq.com

Tengfei Shi, e-mail: stf0623@163.com

Lai Li, e-mail: 920809351@qq.com

Haiqun Cao, e-mail: haiquncao@163.com

Linsheng Yu, e-mail: yulinshengahau@163.com

Supplementary Tab. 1 Sequences filtering statistics.

Sample	input	filtered	percentage of input passed filter	denoised	percentage of input denoised	non-chimeric	percentage of input non-chimeric
BO1	80283	72189	89.9%	71273	88.78%	71273	88.78%
BO2	73448	65137	88.7%	64356	87.62%	64356	87.62%
BO3	77951	67755	86.9%	66976	85.92%	66976	85.92%
PO1	80935	65194	80.1%	64526	79.73%	64526	79.73%
PO2	81113	64876	80.0%	63974	78.87%	63974	78.87%
PO3	73197	65623	89.7%	64731	88.43%	64731	88.43%
MO1	75507	68104	90.2%	67196	88.99%	67196	88.99%
MO2	79810	70935	88.9%	70146	87.89%	70146	87.89%
MO3	63767	57882	90.8%	56890	89.22%	56890	89.22%
LO1	79225	63682	80.4%	62947	79.45%	62947	79.45%
LO2	71039	62006	87.3%	61276	86.26%	61276	86.26%
LO3	72994	58384	80.0%	57774	79.15%	57774	79.15%
EO1	81110	65577	80.8%	64779	79.875	64779	79.87%
EO2	79645	68894	86.5%	68039	85.43%	68039	85.43%
EO3	74631	63278	84.8%	62378	83.58%	62378	83.58%
AO1	75924	63128	83.1%	62344	82.11%	62344	82.11%
AO2	78156	61669	78.9%	60991	78.04%	60991	78.04%
AO3	75432	62867	83.3%	62164	82.41%	62164	82.41%

Input – initial number of sequences. Filtered – number of reads after removing low-quality data. Denoised – number of reads after removing data considered as noise. Non-chimeric – number of sequences after chimera removal.
